# Cholera outbreak in a fishing village in Uganda: a case study

**DOI:** 10.11604/pamj.supp.2018.30.1.15268

**Published:** 2018-05-17

**Authors:** Bao-Ping Zhu, Gerald Pande, Benon Kwesiga, Alex Riolexus Ario

**Affiliations:** 1 Center for Global Health, Centers for Disease Control and Prevention, Kampala, Uganda; 2 Uganda Public Health Fellowship Program, Kampala, Uganda

**Keywords:** Cholera, outbreak investigation, Uganda

## Abstract

In June 2015, the District Health Officer of Kasese District, southwestern Uganda reported an outbreak of cholera in a fishing village. Two fellows of the Uganda Public Health Fellowship Program – Field Epidemiology Track conducted an investigation to verify the existence of an outbreak, determine the mode of transmission, and recommend control measures. This case study describes that investigation, which teaches the steps in an outbreak investigation and the details in each step, what needs to be done in each step to achieve the objectives of the investigation, and what might be the common pitfalls during an outbreak investigation. This case study can be used to teach the fundamental principles of an outbreak investigation and use of Epi Info for outbreak analysis. The audience are field epidemiologists at various levels in different settings.

## How to use this Study

**General instructions:** ideally, 1 or 2 instructors facilitate the case study for 8 to 15 participants in a classroom or conference room. After a brief introduction about the case study, the instructor asks a participant to read aloud a paragraph or two, going around the room and giving each participant a chance to read. After the participant has finished reading a question, the instructor directs all participants to perform calculations, construct graphs, or engage in a discussion of the answer. Sometimes, the instructor can split the class to play different roles, conduct calculations on different parts of a question, or take different sides in answering the question. As a result, participants enrich their learning experience.

**Audience:** FETP trainees (intermediate or advanced levels), public health or surveillance officers at national or subnational levels, Master of Public Health (MPH) students at schools of public health, and other public health officials interested in learning how to conduct outbreak investigations.

**Prerequisites:** case study participants should have received lectures and training on fundamentals of biostatistics, fundamentals of epidemiology, outbreak investigation and Epi Info 7.

**Materials needed:** flip charts, marker set: one per 8-10 participants; Laptop computers with Microsoft Office Suite and Epi Info 7 pre-installed; Projector, projection screen (or substitutes), and LASER pointer

**Level of training and associated public health activity:** Intermediate to Advanced

**Time required:** 2-3 hours

**Language:** English

## Competing interest

The authors declare no competing interest.
